# Combined treatment with niclosamide and camptothecin enhances anticancer effect in U87 MG human glioblastoma cells

**DOI:** 10.18632/oncotarget.28227

**Published:** 2022-05-05

**Authors:** Laura Valdez, Benxu Cheng, Daniela Gonzalez, Reanna Rodriguez, Paola Campano, Andrew Tsin, Xiaoqian Fang

**Affiliations:** ^1^Department of Molecular Science, School of Medicine, University of Texas Rio Grande Valley, Edinburg, TX 78539, USA; ^*^These authors contributed equally to this work

**Keywords:** glioblastoma, niclosamide, camptothecin, cancer, chemotherapy

## Abstract

Glioblastoma multiforme (GBM) is one of the deadliest cancers of the brain. Its ability to infiltrate healthy brain tissues renders it difficult to remove surgically. Furthermore, it exhibits high rates of radio- and chemoresistance, making the survival rates of patients with GBM poor. Therefore, novel effective therapies for GBM remain urgently in demand. Niclosamide is an anti-helminthic drug and recently it has been receiving attention due to its reported anticancer effects in cancer models, including GBM. Furthermore, camptothecin (CPT) is a naturally-occurring alkaloid and has been previously reported to be a potential chemotherapeutic agent by targeting the nuclear topoisomerase I. In the present study, the possible combined chemotherapeutic effects of niclosamide and CPT on the human glioblastoma cell line U87 MG was investigated by MTT assay and western blot analysis. Niclosamide exhibited synergistic activities with CPT to suppress the proliferation of U87 MG cells. Additionally, niclosamide suppressed cell proliferation and induced cell death mainly by triggering ER stress and autophagy, whilst CPT induced cell apoptosis mainly through p53-mediated mitochondrial dysfunction and activation of the MAPK (ERK/JNK) pathways. Overall, these findings suggest that co-administration of niclosamide and CPT may provide a novel therapeutic treatment strategy for GBM.

## INTRODUCTION

Glioblastoma multiforme (GBM) is the most common and aggressive form of brain malignancy in the central nervous system (CNS) [1–3], of which an effective pharmacological therapeutic treatment strategy remains unavailable [1–3]. GBM exhibits high rates of chemo- and radioresistance and potent capabilities to infiltrate healthy brain tissues, rendering it difficult to be removed surgically. Currently available treatment methods for GBM involves combining surgery with chemotherapy. However, the overall survival rate for this disease has not improved to a satisfactory level over the past three decades. One of the main reasons of this is that GBM tumors have high incidence of recurrence, which frequently lead to mortality with a median survival of only 1–3 years from diagnosis [4, 5]. Therefore, development of novel and effective therapies are urgently in demand. One strategy to improve the efficacy of anti-cancer treatments and/or circumvent chemoresistance is to simultaneously disrupt multiple known oncogenic signaling pathways using multiple-target therapeutics. Over the past number of years, various combinations of chemotherapeutic agents have been identified to be viable therapeutic options that outperform monotherapy [6–8]. Therefore, adequate selection of active drugs and understanding their corresponding mechanism in combination will facilitate the development of additional effective chemotherapeutic strategies that can be used for GBM treatment.

Niclosamide is a Food and Drugs Administration-approved oral anti-helminthic drug that has been applied for ~50 years for treating tapeworm infections. Studies over the past decade have demonstrated that niclosamide is also a promising chemotherapeutic agent [9, 10]. In addition, a number of studies have reported that niclosamide can exert potent anti-proliferative activities whilst inducing cytotoxicity in a broad spectrum of cancer cells, including head and neck cancer [11, 12], non-small cell lung cancer [11], thyroid cancer [13], prostate cancer [11], colon cancer [14], ovarian cancer [15], acute myelogenous leukemia [16], breast cancer [17] and osteosarcoma [18]. It has been previously demonstrated that niclosamide can effectively suppress the proliferation/growth of different types of cancers both *in vitro* and *in vivo*. This was mediated by triggering apoptosis, oxidative stress and inhibiting the activities of key signaling pathways, including PI3K/AKT [19], Wnt/β-catenin [20, 21], mTOR [22], NF-κB [16] and STAT3 [11, 19, 23–25]. Furthermore, previous studies have identified a multitude of anticancer agents that when combined with niclosamide, can inhibit tumor growth. In particular, the combination of niclosamide with bicalutamide was found to inhibit the growth of enzalutamide-resistant prostate cancer tumors, suggesting that it can be used to treat advanced prostate cancer [26]. Additionally, niclosamide was observed to synergize with sorafenib to suppress renal cell carcinoma cell proliferation and survival [27]. The combination of niclosamide with enzalutamide was also documented to significantly induce cell apoptosis whilst inhibiting cell proliferation, colony formation, cell migration and invasion. In addition, niclosamide was demonstrated to reverse enzalutamide resistance in prostate cancer cells [28]. Oh et al. [29] recently reported that combined treatment with both niclosamide and temozolomide can significantly reduce cell viability, stemness and suppress the invasive capabilities of GBM tumorspheres [29]. In our previous study, it was also shown that niclosamide can promote cytotoxicity in human glioblastoma cells by downregulating the pro-survival PI3K/AKT, STAT3 and Wnt/β-catenin signal pathways. In addition, niclosamide treatment was found to increase protein ubiquitination, endoplasmic reticulum (ER) stress and autophagy in human glioblastoma cells [19].

Although the anti-cancer activity of niclosamide and its combination with several anti-cancer agents have already been reported in various cell models, studies exploring its potential properties whilst in combination with chemotherapeutic agents in glioma remain limited. Therefore, in the present study, the possible anticancer activity of niclosamide in combination with a previously identified chemotherapeutic anticancer agent camptothecin (CPT) in U87 MG cells was examined. CPT is a naturally-occurring alkaloid derived from the plant *Camptotheca acuminata* that has been reported to exert a novel mechanism of action of targeting the nuclear enzyme topoisomerase I [30]. Previous studies have revealed that CPT and its derivatives, including topotecan (Hycamptin) and irinotecan (Camptosar), display broad antitumor activities against various types of tumor cells both *in vitro* and *in vivo*. Specifically, they have been demonstrated to exhibit anticancer activities against lung cancer [31], gastric cancer [32], hepatocarcinoma [33], esophageal cancer [34], colorectal cancer [35] and breast cancer [36]. In addition to inducing apoptosis in various cancer cell types [37–39], CPT and its derivatives have also been reported to induce senescence in colon [38] and lung cancer cells [40].

Although CPT and its derivatives have been shown to demonstrate potent antitumor activities, chemoresistance to the CPT family of drugs frequently occurs [31, 41, 42]. Furthermore, CPT treatment can result in severe adverse effects in patients, which worsens the therapeutic outcome and makes bone marrow transplantation a common medical practice following CPT treatment [31, 41, 42]. Strategies proposed to resolve these issues of overcoming chemoresistance, minimizing severe adverse effects whilst enhancing efficacy are to design appropriate combinatorial therapeutic methods involving CPTs alongside other anticancer agents capable of targeting a multitude of cellular pathways [42]. Therefore, understanding the underlying mechanism of combination drug action will facilitate the development of novel effective chemotherapeutic methods.

In the present study, the effects of niclosamide and CPT treatment combined on the physiology of the human glioblastoma cell line U87 MG was investigated. Niclosamide, CPT and niclosamide and CPT combination treatment were applied to U87 MG cells. The cell viability and the effects of niclosamide and CPT on the human glioblastoma cells were revealed by MTT assay and western blot analysis. Our findings suggest that combined niclosamide and CPT treatment can be considered as a promising strategy for the treatment of GBM in the future.

## RESULTS

### Effects of niclosamide, CPT and their combination on cell viability and morphological changes in U87 MG cells

**Figure 1:**
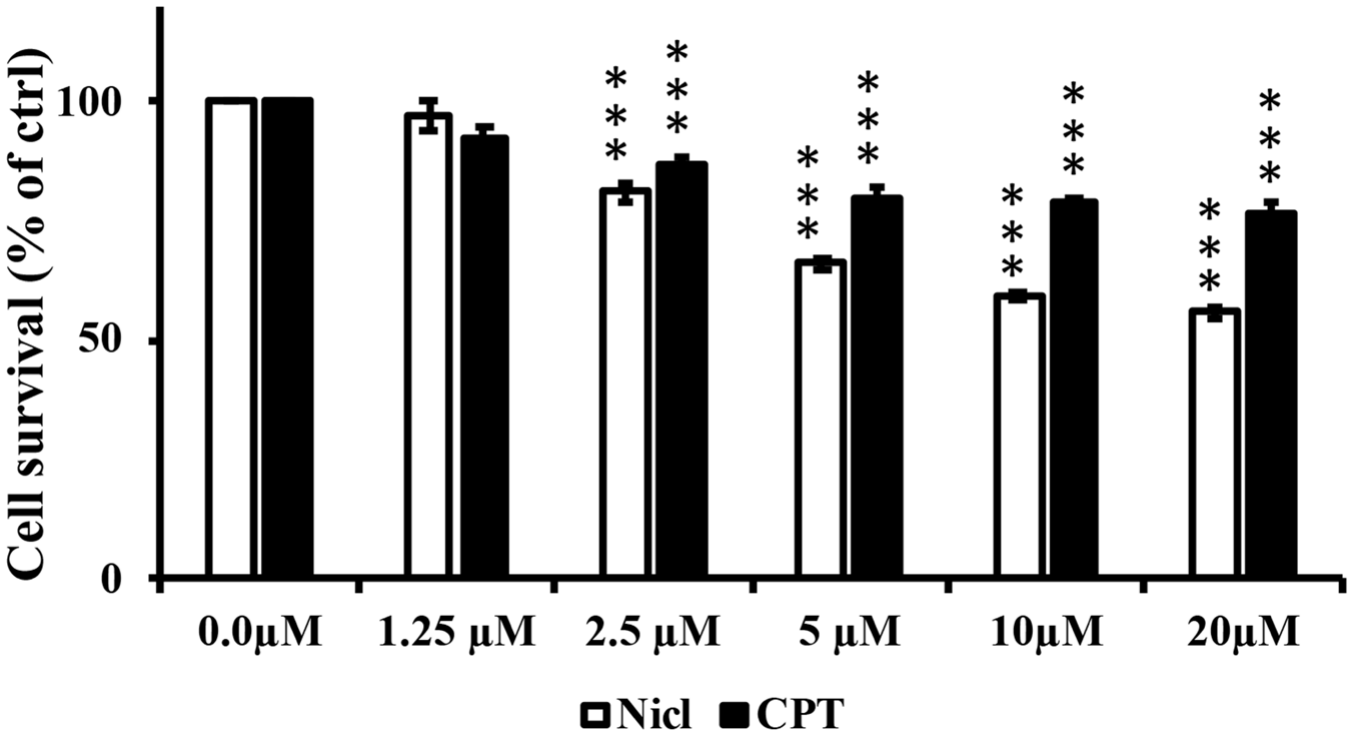
Niclosamide (Nicl) and CPT treatments reduce U87 MG cell viability. U-87 MG cells were treated with the indicated concentrations of niclosamide and CPT for 48 hours. Cell viability was determined by MTT assay. Mean ± S.E.M from five replicates. ^***^ *p* < 0.001 vs. control.

The individual effects of niclosamide or CPT on U87 MG cell viability were first examined. U87 MG cells were treated with different concentrations of niclosamide and/or CPT (0–20 μM) for 48 h. The number of live cells was then measured using MTT assay. As shown in Figure 1, marked inhibitory effects on the survival and viability of U87 MG cells were observed in a dose-dependent manner compared with those in the control group. Based on the results of this initial MTT assay, the doses of 5 μM were selected for both niclosamide and CPT for use in subsequent combinatorial experimentation. The effects of niclosamide and CPT combined on cell morphology and viability were then assessed by comparing with those after treatment with either agent individually.

**Figure 2:**
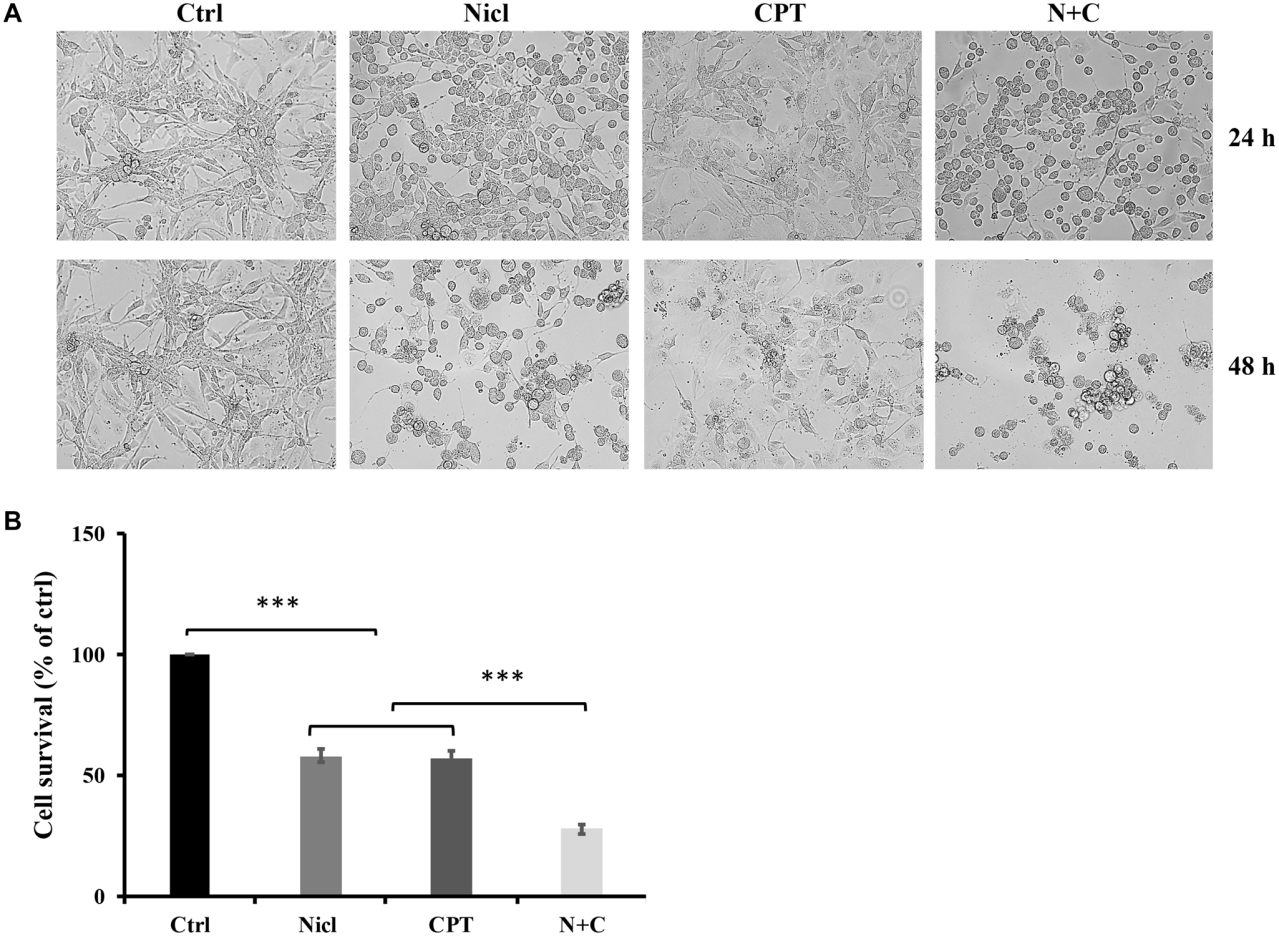
Niclosamide and CPT treatments induce a change in U-87 MG cell morphology and a significant reduction in cell viability. (**A**) Representative images of morphology of U87 MG cells were treated with 5 μM niclosamide (Nicl), 5 μM CPT, and their combination for 24 and 48 hours. Cells were imaged by phase-contrast microscopy. (**B**) U87 MG cells were treated with 5 μM niclosamide (Nicl), 5 μM CPT, and their combination for 48 hours. Cell viability was determined by MTT assay. Data represent the mean ± S.E.M of at least three independent experiments. ^***^ *p* < 0.001.

As shown in Figure 2A, cell morphology was more altered by both treatments in combination compared with that after treatment with either agent alone. Control cells showed high confluency in the cell monolayer, whereas treatment with either 5 μM niclosamide or 5 μM CPT alone induced notable morphological changes. After treatment with niclosamide, a large proportion of cells exhibited spherical shapes, reduced cell volumes and cell densities. By contrast, different morphological changes were observed after the cells were treated with CPT. CPT exposure induced senescence-like phenotypes, including flattened cell shape with enlarged cell bodies and intensive blebbing. The combined treatment with niclosamide and CPT induced clear morphological changes, where the cells tended to be rounder in shape and smaller in size. In addition, an increased number of cells were observed to be detached from the surface, resulting in lower cell densities. Taken together, these observed changes suggest that the co-treatment of niclosamide and CPT exerted marked negative effects on U87 MG cell morphology. In terms of the cell viability assay, U87 MG cells were treated with 5 μM niclosamide and/or 5 μM CPT for 48 h. Treatment with either niclosamide or CPT alone was found to reduce cell viability (Figure 2B). By contrast, co-treatment with niclosamide and CPT significantly reduced cell viability compared with that after treatment with either agent alone (Figure 2B), implicating suppressive effects of niclosamide and CPT co-treatment on the viability of U87 MG cells. Following this observation of the effects of niclosamide and CPT co-treatment, analysis was subsequently performed to investigate the mechanism underlying the effects of niclosamide and CPT on cell survival.

### Effect of niclosamide and CPT on protein ubiquitination

**Figure 3:**
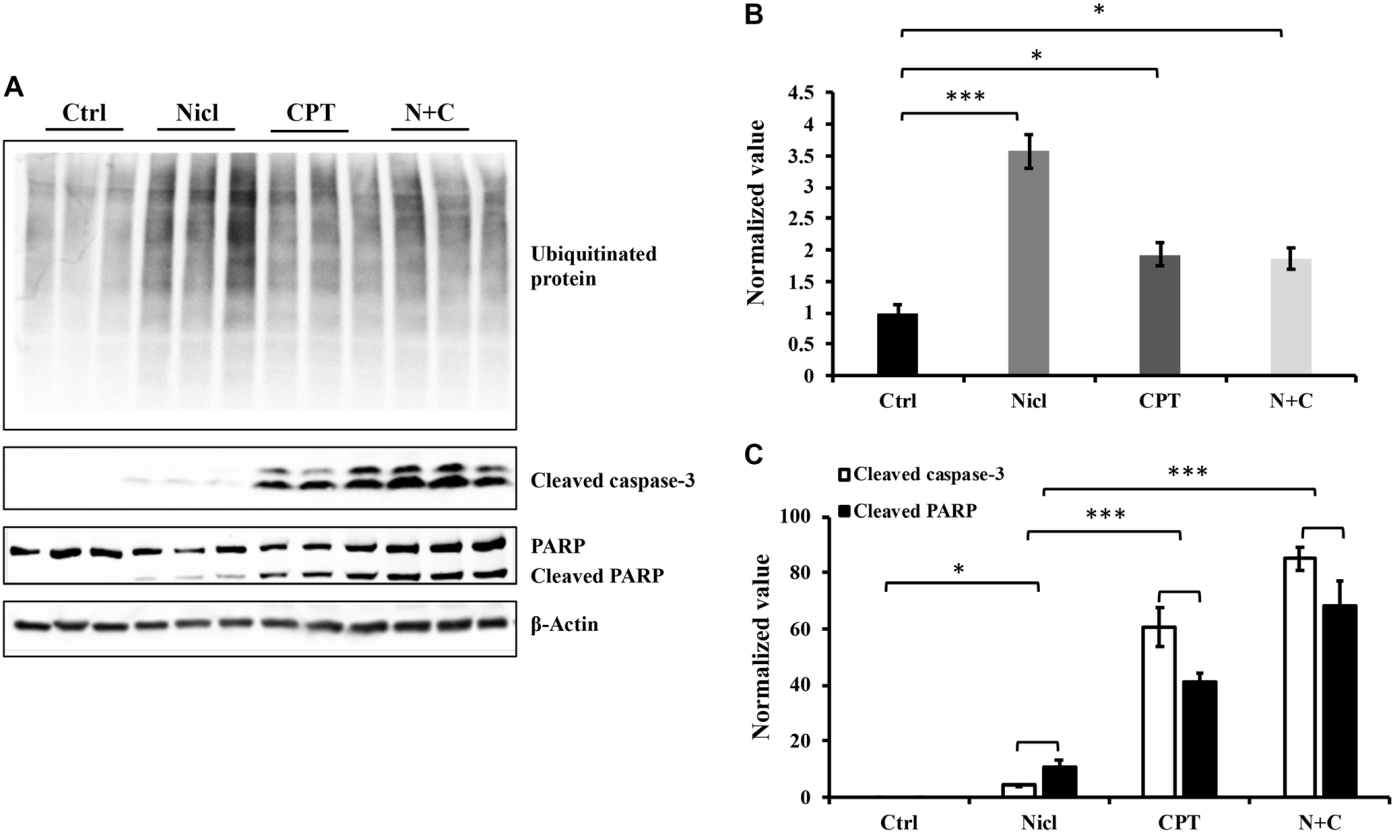
Niclosamide and CPT induce protein ubiquitination respectively and synergistically enhance caspase-3 and PARP cleavage. The cell lysates were prepared from U87 cells treated with either 5 μM niclosamide (Nicl), or 5 μM CPT alone, or with combined treatment for 24 hours and resolved by SDS-PAGE and then immunoblotted with antibodies specific for ubiquitin, cleaved PARP, and cleaved caspase-3 (**A**). β-Actin was used as the loading control. The protein levels from Western blot were quantified by densitometry. (**B**) Ubiquitinated proteins. (**C**) Cleaved PARP and cleaved caspase-3. Data represent the mean ± S.E.M. ^*^ *p* < 0.05; ^***^ *p* < 0.001.

The ubiquitin-proteasome system has emerged as an attractive novel cancer chemotherapeutic target. Pre-clinical studies have previously shown that inhibition of the proteasome pathway using small-molecule proteasome inhibitors resulted in cell cycle arrest and apoptosis in cancer cell lines and murine models of cancer [43]. In addition, our previous study demonstrated that niclosamide functioned as a proteasome inhibitor to potently increase protein ubiquitination and induce apoptosis in U87 MG cells [19]. Therefore, in the present study, the effects of CPT on protein ubiquitination in U87 MG cells were evaluated. The quantity of ubiquitinated proteins were measured by western blotting using an antibody against ubiquitin. Our results confirm our previous finding that niclosamide promoted the accumulation of ubiquitinated proteins (Figure 3A). CPT also promoted protein ubiquitination in U87 MG cells, albeit to a lesser extent compared with niclosamide, although the CPT-induced ubiquitinated protein levels are significantly higher compared with those in the control group (Figure 3B). However, the combined treatment of niclosamide and CPT did not increase the levels of protein ubiquitination compared with that mediated by CPT treatment alone.

### Niclosamide and CPT induces cell apoptosis in a p53-dependent manner

Activation of caspase is a typical feature of apoptotic cell death. Caspase-3 is considered to be a key executioner caspase that serves a central role in mediating apoptotic responses [44]. The majority of chemotherapeutic agents induce tumor cell death through the caspase activation process. Activation of caspase-3 leads to the cleavage of poly-(ADP ribose) polymerase (PARP), which is considered to be another hallmark of apoptosis. Our previous study demonstrated that niclosamide is able to activate caspase-3 and subsequent PARP cleavage in a dose-dependent manner [19]. To determine the combined effects of niclosamide and CPT on apoptosis and cell death in U87 MG cells, caspase-3 activation and PARP cleavage was measured through western blot analysis. After incubation with niclosamide and/or CPT for 24 h, cell lysates were examined for protein expression. As shown in Figure 3A, treatment with either niclosamide or CPT alone led to the increased activation of caspase-3 and PARP cleavage. However, compared with niclosamide, CPT was the more potent activator of both caspase-3 and PARP cleavage. This suggests that CPT mainly causes cell death in a caspase-dependent manner in U87 MG cells. Further quantitative analysis revealed that combined treatment with CPT and niclosamide caused significant increases in the cleavage of caspase-3 and PARP compared with those after either drug treatment alone (Figure 3C).

**Figure 4:**
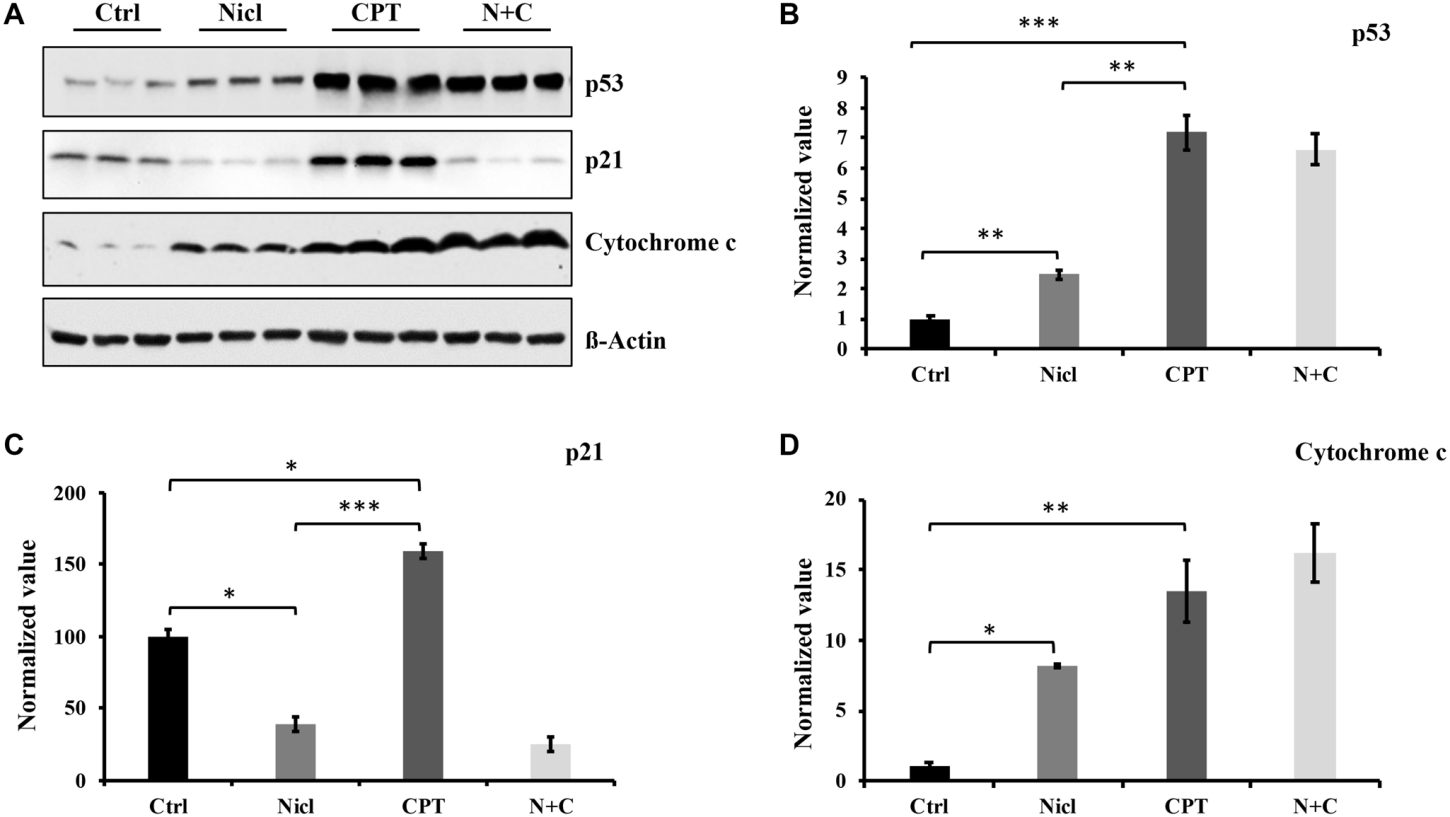
Niclosamide enhances p53 protein and cytochrome c expression but suppresses p21 expression. CPT strongly promotes p53, p21, and cytochrome c expression. (**A**) U87 MG cells were treated with either 5 μM niclosamide (Nicl), 5 μM CPT, or a combination of both for 24 hours. Thereafter, the cells were collected, and the cell lysate was prepared for Western blot analysis. The proteins were resolved by SDS-PAGE and immunoblotted with specific antibodies against p21, p53, and cytochrome c. β-Actin was used as the loading control. (**B**) p53 protein expression levels. (**C**) p21 protein expression levels. (**D**) Cytochrome c protein expression levels. Data represent the mean ± S.E.M of three replicates. ^*^ *p* < 0.05; ^**^ *p* < 0.01; ^***^ *p* < 0.001.

p53 is a well-known tumor suppressor protein and that is found to be mutated in ~50% of all human cancers [45]. In addition, p53 mutations have been found in 87% of all GBM cases [45]. It has also been reported that various types of cells treated with CPT can undergo p53-dependent apoptosis [46]. p53 activation has been previously shown to promote the activation of caspases-3 and -7, causing apoptosis in human glioblastoma cells [47]. By contrast, p21 is a cell cycle inhibitor that serves an important role in cell cycle arrest and early senescence. In a number of cell types, although p53 activation leads to the induction of p21, p21 can also be regulated independently of p53 activity [48]. To measure the expression of p53 and p21 proteins following combined treatment with niclosamide and CPT, U87 MG cells were treated with either niclosamide and/or CPT for 24 h. Western blot analysis was then performed to detect p53 and p21 protein expression. As shown in Figure 4A and 4B, basal expression levels of p53 and p21 were relatively low in U87 MG cells. The expression level of p53 in niclosamide-treated cells showed a weak but significant increase compared with that in the control. However, the expression level of p53 in CPT-treated cells was potently and significantly increased compared with that in the niclosamide-treated cells. p53 expression after the co-treatment of niclosamide and CPT remained comparable to that after CPT treatment alone. These data are consistent with those reported in a previous study reporting that CPT is a strong p53 protein inducer in a variety of cell lines [38]. Since p21 is one of the downstream targets of p53, p21 expression was also examined in niclosamide- and/or CPT-treated U87 MG cells. As shown in Figure 4A and 4C, U87 MG cells exposed to CPT for 24 h exhibited substantially higher levels of p21 compared with that in control, which was positively associated with those of p53 expression. However, niclosamide exposure not only reduced the basal levels of p21 expression compared with that in control, but also completely abolished the CPT-induced p21 activation as shown in cells co-treated with niclosamide and CPT.

Apoptosis induced by the mitochondrial apoptotic pathway typically involves the activation of caspases. Since both niclosamide and CPT can induce activation of caspase-3 and PARP cleavage, cytochrome *c* expression was next evaluated after exposure to niclosamide and CPT. As shown in Figure 4A and 4D, cytochrome *c* expression was significantly increased in U87 MG cells following treatment with either niclosamide or CPT, respectively. In addition, co-treatment with niclosamide and CPT synergistically increased cytochrome *c* expression compared with either treatment alone. Overall, the levels of cytochrome *c* expression in niclosamide- and CPT-treated cells associated positively with the expression levels of p53, suggesting that niclosamide and CPT can induce U87 MG cell apoptosis in a p53-dependent manner.

### Activation of MAPK (ERK/JNK) signaling enhances U87 cell toxicity

**Figure 5:**
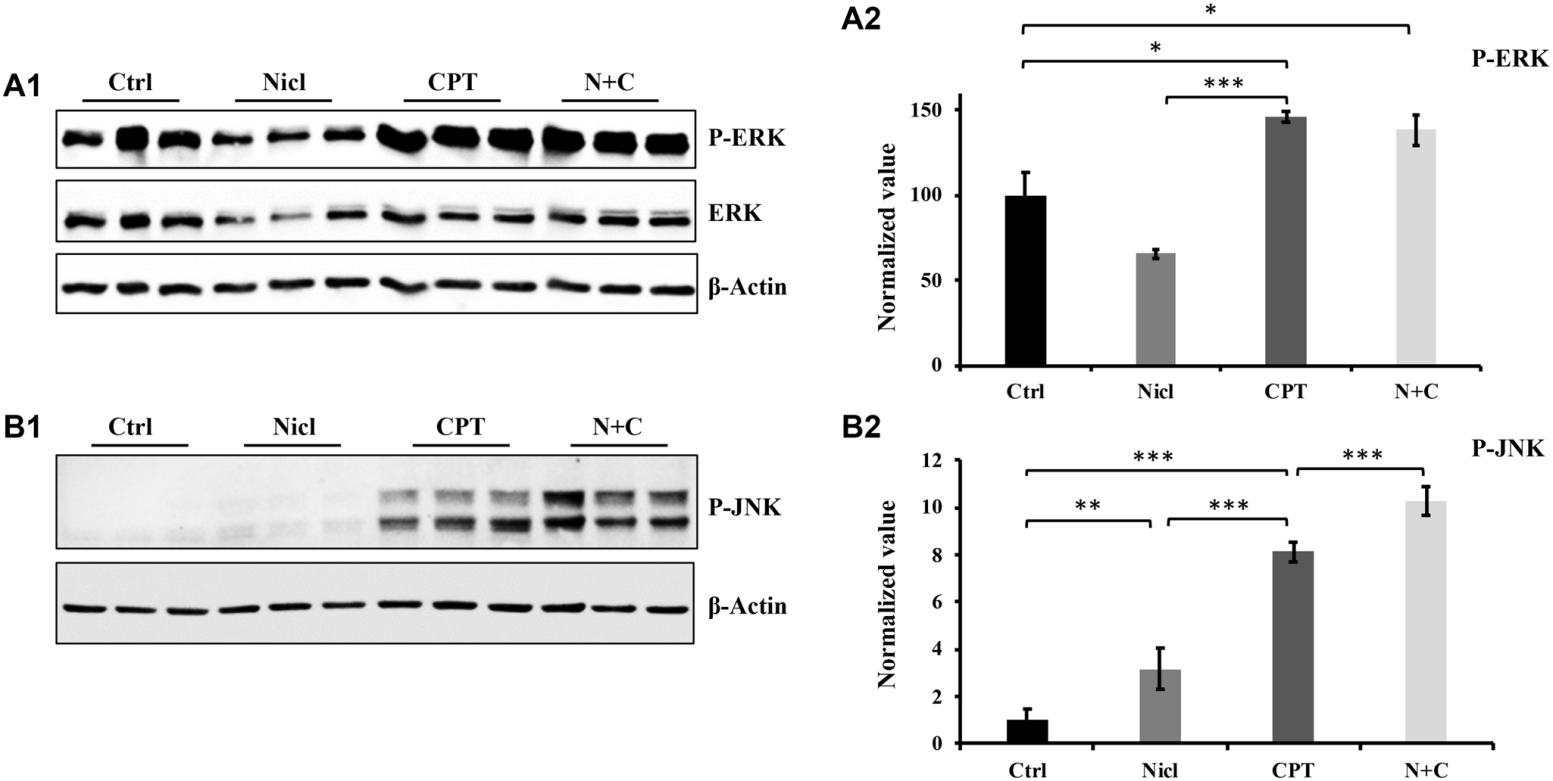
The effect of niclosamide and CPT on MAPK pathway. (**A1**) Niclosamide (Nicl) inhibits ERK phosphorylation, whereas CPT induces abundant ERK phosphorylation. (**A2**) Densitometry analysis of p-ERK phosphorylation. (**B1**) Niclosamide slightly upregulates JNK phosphorylation compared with control, whereas CPT dramatically increases JNK phosphorylation. The combined treatment of the two synergistically upregulates JNK phosphorylation. (**B2**) Densitometric analysis of p-JNK. Total protein was isolated from U87 MG cells treated with 5 μM niclosamide, 5 μM CPT, or their combination for 24 hours. Lysates were resolved by SDS-PAGE and immunoblotted with antibodies, as indicated in the figures. ^*^ *p* < 0.05, ^**^ *p* < 0.01, and ^***^ *p* < 0.001.

MAPKs serve important roles in a diverse range of cellular processes, especially apoptosis and cell survival. There are three major families of MAPKs: ERK, JNK and p38 MAPK (p38). Upregulation of JNK signaling has been extensively reported to be associated with increased cytotoxicity in various cell lines, including glioblastoma [49, 50]. In addition, CPT has been reported to activate the MAPK pathway to trigger apoptosis in human gastric cancer cell lines [51]. CPT has also been found to activate JNK and induce oxidative stress in human non-small cell lung cancer cells [31]. Our previous study has shown that niclosamide was able to inhibit the activation of ERK in U87 MG cells [19]. In the present study, the regulation of ERK and JNK signaling was focused in the U87 MG cells. First, the effects of niclosamide and/or CPT on ERK signaling regulation were assessed. As shown in Figure 5A, ERK expression and phosphorylation were downregulated in cells treated with niclosamide alone, which was consistent with findings from our previous study [19]. By contrast, ERK phosphorylation was drastically increased in cells treated with CPT for 24 h. After niclosamide and CPT co-treatment, the CPT-induced ERK phosphorylation remained unchanged (Figure 5A).

Next, the potential effects of niclosamide and CPT on JNK signaling were investigated. Regulation of JNK appeared to be different from ERK, since both niclosamide and CPT induced JNK phosphorylation (Figure 5B). Niclosamide weakly but significantly increased JNK phosphorylation compared with that in the control group (Figure 5B). JNK phosphorylation was also effectively stimulated after CPT treatment for 24 h. Furthermore, co-treatment with both CPT and niclosamide significantly enhanced JNK phosphorylation in U87 MG cells compared with that after either individual treatment alone (Figure 5B). Therefore, the phosphorylation profile of JNK was consistent with the aforementioned western blotting data regarding apoptotic events, namely caspase-3 activation, PARP cleavage and cytochrome *c* expression (Figures 3 and 4).

**Figure 6:**
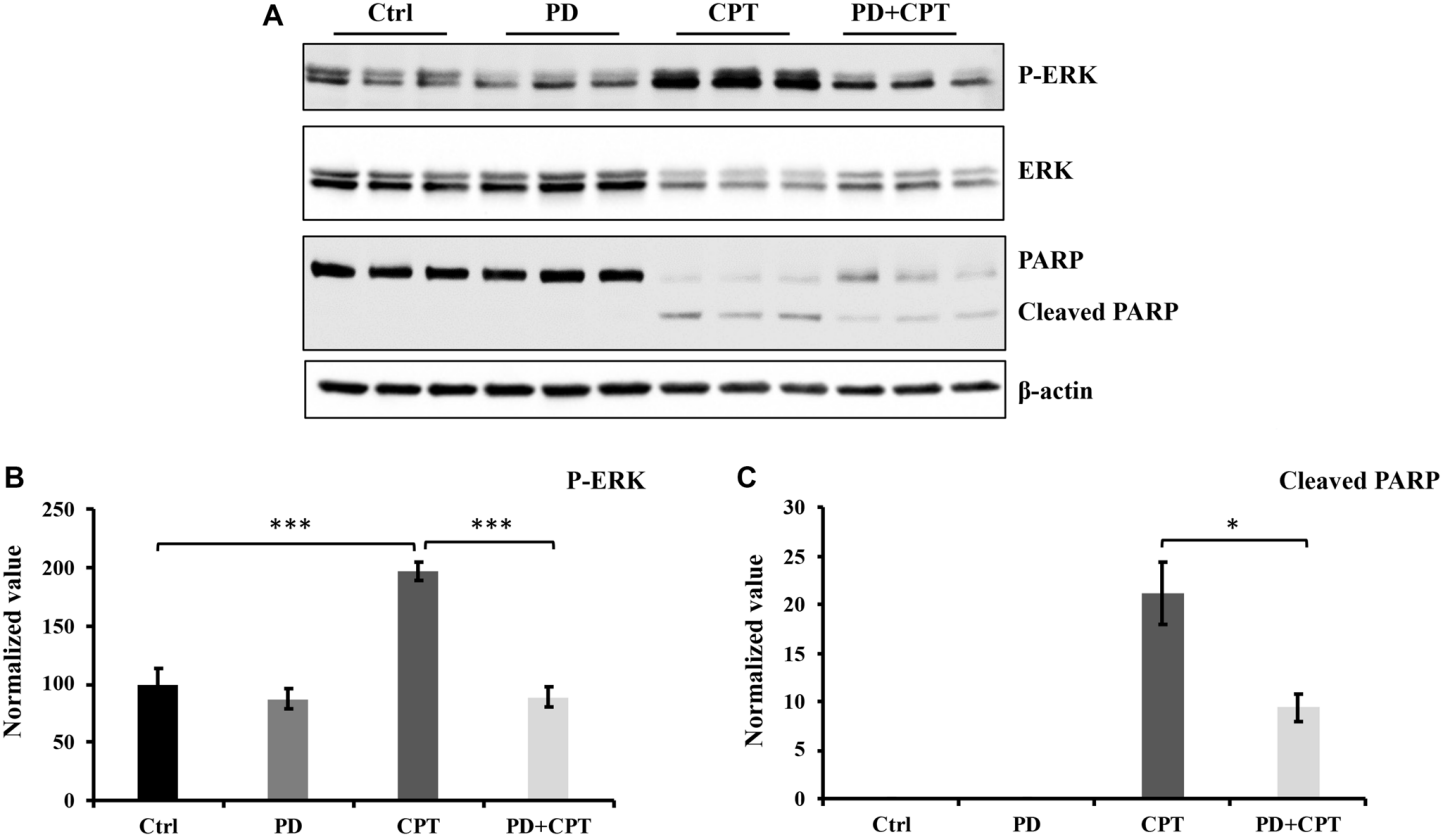
The activation of ERK by CPT has no significant effect on cell apoptosis. U87 MG cells were pretreated with 20 μM ERK signaling pathway inhibitor PD98059 (PD) for 1 hour and followed by exposure of 5 μM CPT for 24 hours. The cells were collected, and cell lysates were prepared for Western blot analysis. Antibodies used were indicated in Figure 8. (**A**) Western blot profile. (**B**) Densitometric values for p-ERK. (**C**) Densitometric value for cleaved PARP. Data represent the mean ± S.E.M of three replicates. ^*^ *p* < 0.05; ^**^ *p* < 0.01; ^***^ *p* < 0.001.

Unlike JNK, activation of ERK can mediate both protective and harmful effects [52–54]. Therefore, the role of CPT on ERK signaling was next investigated in U87 MG cells. To determine the effects of p-ERK1/2 on apoptosis induction by CPT in U87 MG cells, the ERK inhibitor PD98059 was used. After the U87 MG cells were cultured to ~80% confluency, they were pre-treated with 20 μM ERK inhibitor PD98059 for 1 h followed by the addition of 5 μM CPT for 24 h. Subsequently, cell lysates underwent western blotting against cleaved PARP to assess the effects of ERK activation on apoptosis. As shown in Figure 6A and 6C, CPT-induced PARP cleavage was significantly reduced after the cells were pre-treated with PD98059. These results suggest that ERK activation partially contributes to CPT-induced apoptosis (Figure 6B).

### CPT induces autophagy and suppresses niclosamide-induced ER stress in U87 MG Cells

**Figure 7:**
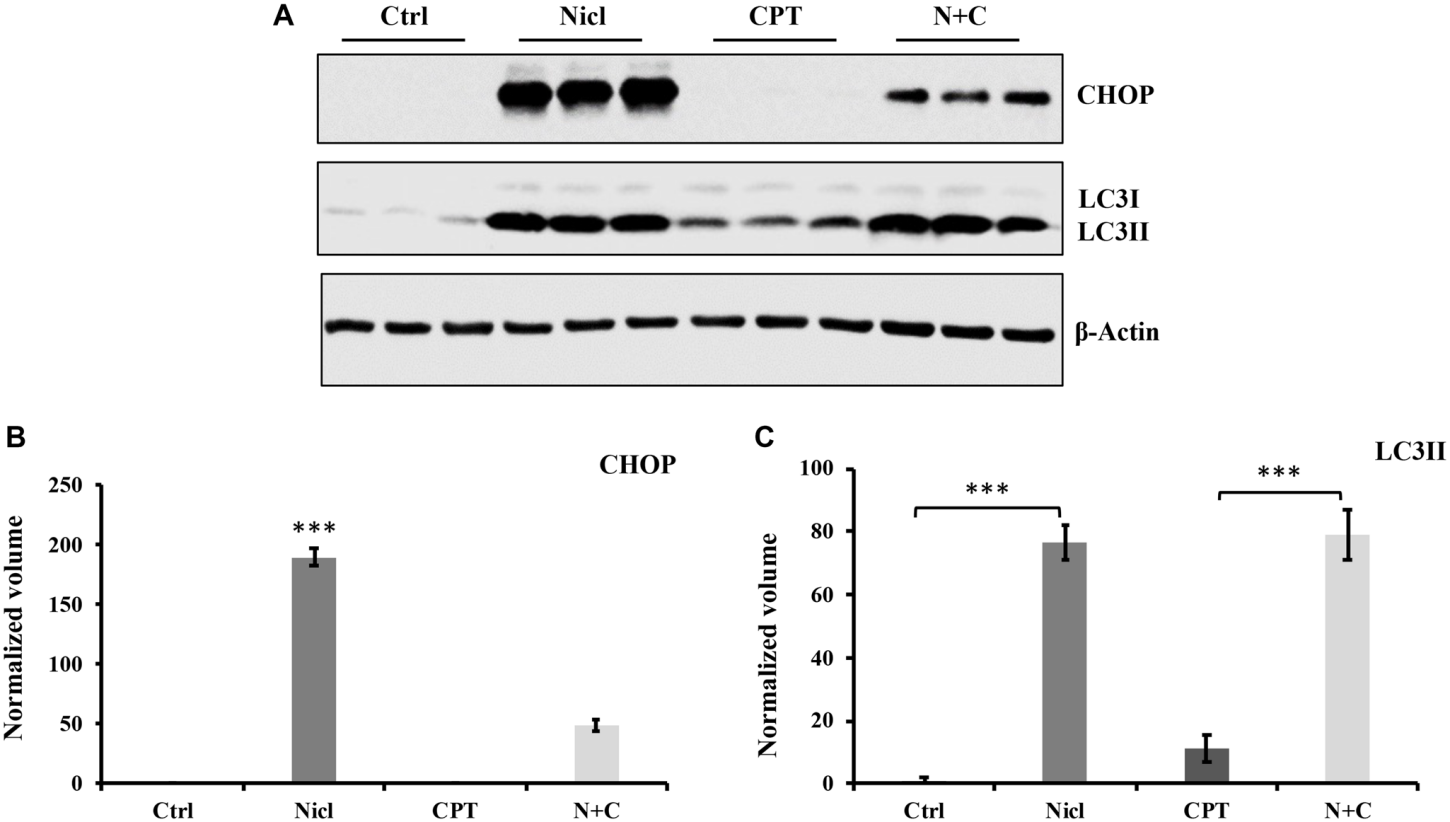
Niclosamide triggers ER stress, whereas CPT inhibits ER response in U-87 MG cells. Both niclosamide and CPT induce the autophagic response in U-87 MG cells. (**A**) U87 MG cells were treated with 5 μM niclosamide (Nicl), 5 μM CPT, or their combination for 24 hours, then collected for lysate preparation. Total cell lysates were resolved on SDS-PAGE and immunoblotted with antibodies specific for CHOP and LC3. β-actin was used as the loading control. (**B**) Relative expression levels of CHOP. (**C**) Relative expression of LC3II. Data represent the mean ± S.E.M of three replicates. ^***^ *p* < 0.001.

Autophagy serves an important role in cell metabolism by degrading intracellular macromolecules and damaged organelles to maintain cell homeostasis [55]. Our previous study reported that niclosamide can stimulate ER stress and autophagic apoptosis [19]. In the present study, the potential effects of CPT on ER stress and autophagy in U87 MG cells were next investigated. ER stress was detected by measuring the expression of CHOP, an ER stress marker. During autophagy induction, LC3I is converted to LC3II, a marker widely used for measuring autophagy. In the present study, autophagy induction was confirmed by measuring LC3II expression. U87 MG cells were first exposed to 5 μM niclosamide and/or 5 μM CPT for 24 h. Thereafter, cell lysates were collected for western blotting. Niclosamide was found to upregulate the expression of CHOP and LC3II (Figure 7A), consistent with findings from our previous study [19]. By contrast, CPT treatment was unable to affect CHOP expression, whereas the increased levels of CHOP expression triggered by niclosamide were significantly reversed by CPT co-treatment (Figure 7B). Furthermore, CPT was detected to modestly but significantly elevate LC3II expression compared with that in the untreated control group (Figure 7C). This suggests that CPT could not stimulate ER stress and even suppress niclosamide-induced ER stress.

### Differential effects of niclosamide and CPT on the expression of cell cycle regulators

**Figure 8:**
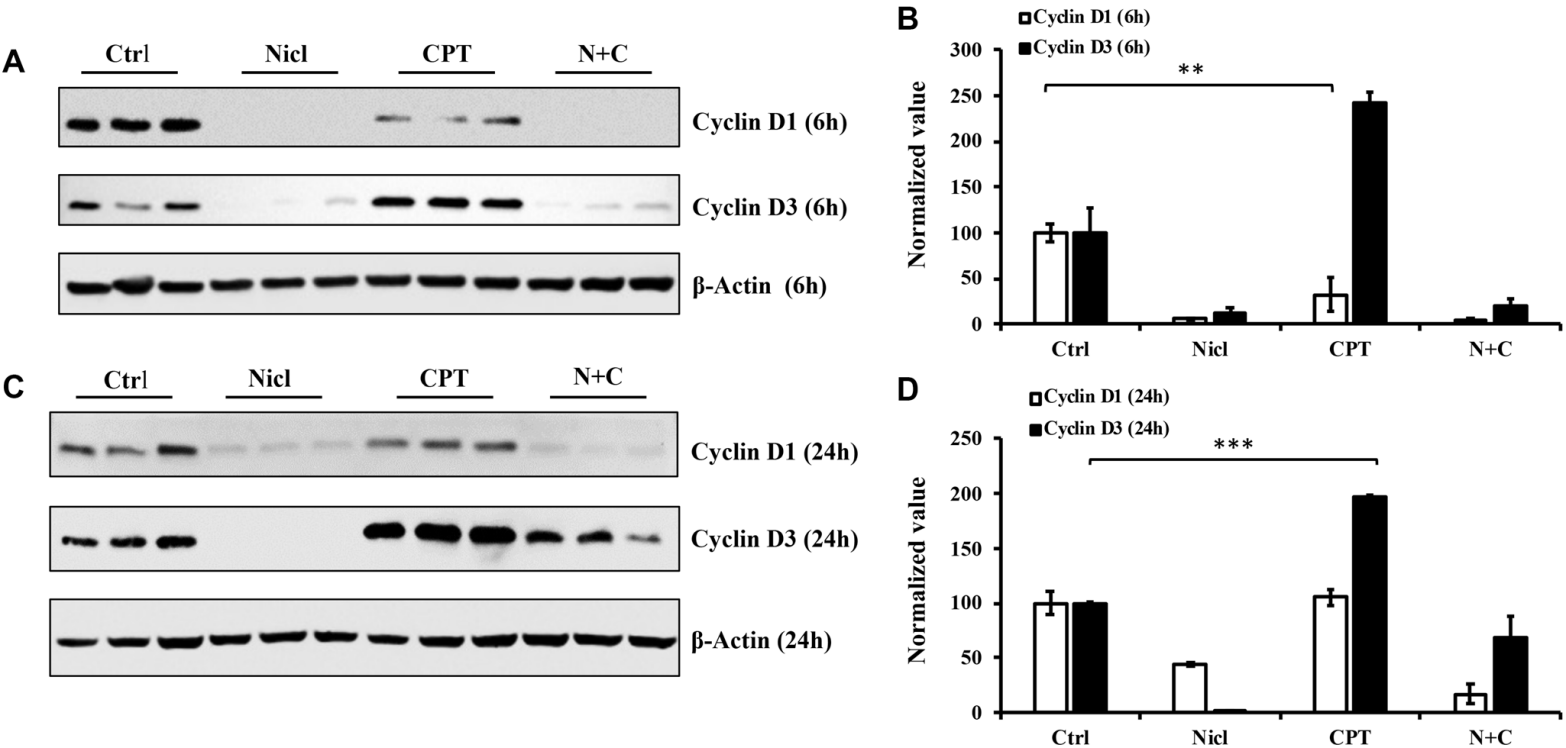
Niclosamide represses cyclin D1 expression, whereas CPT markedly enhances cyclin D3 expression. The cell lysates were prepared from U87 MG cells treated with either 5 μM niclosamide (Nicl), 5 μM CPT, or their combination for 6 and 24 hours. Samples were resolved by SDS-PAGE and immunoblotted with antibodies specific for cyclin D1 and cyclin D3. β-Actin was used as the loading control. (**A**) Cell lysates from cells treated for 6 hours. (**B**) Statistical analysis of D1 and D3 expression of 6-hour treated cells. (**C**) Cell lysates from cells treated for 24 hours. (**D**) Statistical analysis of cyclin D1 and D3 expression of 24-hour treated cells. Data represent the mean ± S.E.M of three replicates. ^*^ *p* < 0.05; ^**^ *p* < 0.001; ^***^ *p* < 0.001.

Cyclins D1 and D3 are important regulators of cell cycle progression from G_1_ to S phases. Both regulators serve as positive growth stimuli for cell cycle progression and cell proliferation. Cyclin D1 and D3 have been previously reported to be proto-oncogenes [56, 57]. In our previous study, it was demonstrated that niclosamide can inhibit cyclin D1 expression in U87 MG cells [19]. In the present study, both cyclin D1 and cyclin D3 protein expression were measured after the exposure of U87 MG cells to 5 μM niclosamide and/or 5 μM CPT for 6–24 h. As shown in Figure 8A, niclosamide potently suppressed both cyclin D1 and cyclin D3 expession following 6 h of treatment. However, unlike niclosamide, CPT only partially inhibited cyclin D1 protein expression whilst significantly enhancing cyclin D3 expression compared with those in the control group (Figure 8A and 8B). Both cyclin D1 and D3 expression were reduced by combined treatment compared with those in the control group. After exposing the cells to niclosamide for 24 h, both cyclin D1 and D3 expression were abrogated, similar to the findings after 6 h of treatment (Figure 8C). However, CPT treatment for 24 h yielded cyclin D1 protein expression levels comparable to those in the control group whilst enhancing cyclin D3 protein expression. In comparison to the 6 h combinatorial treatment, cyclin D1 expression was almost completely suppressed after 24 h of combined CPT and niclosamide treatment (Figure 8A and 8C). Furthermore, cyclin D3 expression was significantly reduced by combined treatment, compared with that in the CPT treatment group (Figure 8D). Taken together, these results suggest that niclosamide had the capability to inhibit both cyclin D1 and D3 expression. By contrast, CPT temporarily inhibited cyclin D1 expression but enhanced cyclin D3 expression. Furthermore, combined treatment inhibited both cyclin D1 and D3 expression in U87 MG cells.

## DISCUSSION

Glioblastomas belong to a family of malignancies in the central nervous system with poor overall survival rates and requires novel and effective therapeutic approaches to tackle. One of the approaches to treat glioblastoma is a combination therapy. Combination therapy refers to a treatment strategy that combines ≥ two therapeutic agents in a single regimen. This type of therapy has been documented to increase efficacy compared with that by monotherapeutic approaches by targeting multiple oncogenic signaling pathways in a synergistic manner. This approach can also reduce drug resistance and drug dosage in addition to lowering the overall cost. It was previously demonstrated that niclosamide can serve as a proteosome inhibitor to stimulate the accumulation of ubiquitinated proteins, in turn inducing apoptosis, ER stress and autophagy. Furthermore, niclosamide has been shown to inhibit a multitude of survival signaling pathways in human glioblastoma U87 MG cells [19]. Therefore, in the present study, these previous findings were extended by investigating the combinatorial effects of niclosamide and CPT treatment on U87 MG cells. Previous studies have shown that niclosamide and CPT are cytotoxic. Furthermore, they exhibit anticancer activity by regulating a range of important cellular mechanisms in various types of cancer cells. The aim of the present study was to investigate the effects and underlying mechanism of niclosamide and/or CPT on the glioblastoma cell line U87 MG.

Our previous findings have shown that niclosamide can induce cytotoxicity in U87 MG cells. In the present study, the potential cytotoxic effects of niclosamide combined with CPT in U87 MG cells was first evaluated. Treatment with either niclosamide or CPT alone inhibited cell viability in a dose-dependent manner (Figure 1), whilst combined treatment of the two decreased cell viability further. In addition, as shown in Figure 2A, distinct morphological changes were observed between niclosamide- and CPT-treated U87 MG cells. After treatment with niclosamide for 48 h, the majority of the cells demonstrated a spherical shape, reduced cell volume and cell density. CPT exposure induced senescence-like cell phenotypes, including flattened and enlarged cell bodies in addition to intensive blebbing, which were consistent with the findings of Morandi et al. [58]. However, the apparent senescent cell morphology disappeared after co-treatment with niclosamide. These results suggest that the cell morphological changes observed after combined treatment were likely to be driven by different mechanisms downstream.

In our present study, CPT treatment, like niclosamide, can lead to cell apoptosis in U87 MG cells, which is also consistent with previous studies, which revealed that CPT and its derivatives can induce apoptosis in different cancer cell lines, including lung cancer [31], human breast cancer MCF-7 [59] and MDA-MB-231 [60] cells and human colon cancer cells [38]. The combination of niclosamide and CPT enhanced cytotoxicity in U87 MG cells further and exhibited significant potentiating effects on the induction of apoptosis, as evidenced by the observed caspase-3 activation and PARP cleavage (Figure 3). Since niclosamide has been previously identified to be a promoter of protein ubiquitination and cell apoptosis in U87 MG cells [19], the effects of CPT treatment on niclosamide-induced protein ubiquitination were next examined. Similar to the effects mediated by niclosamide, administration of CPT also resulted in the accumulation of ubiquitinated proteins. Although the level of ubiquitinated proteins is significantly higher compared with that in the control group, CPT-induced ubiquitinated protein levels were substantially less potent compared with those in niclosamide-induced cells (Figure 3). The effect of CPT on the accumulation of protein ubiquitination has not previously been reported in U87 MG cells. The mechanism of CPT-induced protein ubiquitination may be unique in human glioblastoma cells. Studies have shown that protein ubiquitination can serve a role in tumor cell proliferation, survival and apoptosis [61]. In the present study, niclosamide- and CPT-induced protein ubiquitination may be one of the mechanisms for the induction of cytotoxicity in U87 MG cells. However, the underlying mechanism of protein ubiquitination following niclosamide and CPT treatment in glioblastoma cells remain questions that require further examination in future studies.

Apart from the ubiquitin-proteasome system, other mechanisms can also be involved in regulating cell apoptosis, such as the p53 pathway. Activated p53 in turn triggers several responses, including cell cycle arrest, senescence, differentiation and apoptosis [62]. In the present study, p53 activation was measured in niclosamide- and/or CPT-treated U87 MG cells. Consistent with previous findings in different cell types [38, 60, 63, 64], CPT strongly enhanced both p21 and p53 protein expression in the U87 MG cell line. By contrast, niclosamide only modestly increased p53 expression compared with that in CPT. In particular, niclosamide treatment almost completely suppressed both basal p21 and CPT-induced p21 expression. This observation suggests that niclosamide and CPT induce cell responses through distinct mechanisms. It has been reported that p53 can enhance cell autophagy, induce cell senescence and apoptosis [62, 65]. In terms of p21, downregulated p21 expression has been reported to induce autophagy whereas sustained p21 activity is required for the induction of senescence [24, 38]. Findings from the present study regarding p53 and p21 suggest that niclosamide may induce cell death by triggering cytotoxic autophagy whilst CPT causes the development of cell senescence. Furthermore, both niclosamide and CPT may ultimately induce cell apoptosis through different mechanism.

Cytochrome *c* serves an important role in cell death. A previous study found that teniposide and various other chemotherapeutic agents can induce dose-dependent increases in the expression of cytochrome *c* upstream of cell death induction [66]. In the present study, cytochrome *c* expression in U87 MG cells treated with niclosamide and/or CPT was also measured by western blotting 24 h after treatment. As shown in Figure 4, niclosamide triggered a small increase in cytochrome *c* expression, whereas CPT markedly increased cytochrome *c* expression. In addition, combined treatment with both drugs promoted cytochrome *c* expression further. The increased level of cytochrome *c* expression is positively associated with the levels of p53 protein expression in the present study, suggesting that p53 is involved in mediating U87 MG apoptosis. p53 and cytochrome *c* expression are also found to be positively associated with caspase-3 activation and PARP cleavage. These findings suggested that niclosamide and CPT can increase the expression of p53 and cytochrome *c*, where high expression levels of cytochrome *c* released into the cytoplasm in turn activate the caspase-3/PARP cascade downstream. Therefore, both niclosamide and CPT are likely to cause cell death through the caspase-dependent mitochondrial pathway by activating cytochrome *c*.

Accumulating evidence suggests that the p53 protein can regulate MAPK signaling pathways in response to cell stress. MAPK signaling pathways, including JNK, p38 and ERK, regulate various cell responses, including proliferation, migration, differentiation and apoptosis. In the present study, significant increases in p53 expression were observed in U87 MG cells treated with niclosamide and/or CPT. Therefore, the effects of niclosamide and/or CPT treatment on ERK and JNK phosphorylation/activation were next evaluated. Consistent with findings from a previous study, niclosamide downregulated ERK expression and activation (Figure 5A1 and 5A2). Furthermore, it was found that niclosamide was able to slightly enhance JNK phosphorylation (Figure 5B1 and 5B2). By contrast, CPT significantly increased both ERK and JNK phosphorylation. This CPT-induced ERK activation can be suppressed by the ERK inhibitor PD98059 (Figure 6). Although niclosamide reduced the basal expression levels of ERK and ERK phosphorylation, it could not influence CPT-induced ERK phosphorylation in U87 MG cells following combined CPT and niclosamide treatment. In addition, niclosamide and CPT co-treatment significantly increased JNK phosphorylation. It has been previously reported that DNA damage induced by cisplatin can activate ERK1/2 and increase p53 protein expression [67], leading to p53-mediated cellular responses [68]. After the U87 MG cells were treated with CPT or both CPT and niclosamide, it is possible that DNA damage was induced, which activated the ERK signaling pathway to upregulate p53 expression and promote apoptosis. A previous study demonstrated that under cellular stress, JNK-mediated phosphorylation can stabilize and activate p53 to promote programmed cell death [69, 70]. Results from the present study are consistent with previous findings reporting that niclosamide and CPT can upregulate JNK phosphorylation, which is in turn positively associated with the findings of increased p53 expression, cytochrome c expression, caspase-3 activation and PARP cleavage in the present study. These results suggest that in U87 MG cells, CPT can induce apoptosis by activating the ERK pathway, whereas both niclosamide and CPT can induce apoptosis by upregulating JNK activation.

ER stress is another key regulator of apoptosis. A number of anti-cancer therapies have been shown to be associated with the induction of ER stress in tumor cells [71]. Therapeutic induction of ER stress-induced apoptosis has been shown to be beneficial for killing cancer cells [72]. Findings from the present study are consistent with the hypothesis that niclosamide treatment triggered ER stress, as evidenced by the elevated CHOP expression observed. CHOP is an ER stress marker that is considered to serve an important role in the induction of apoptosis [73]. The present study suggests that niclosamide-induced ER stress served a key role in inducing cytotoxicity and cell death. However, CPT not only failed to induce ER stress, but also reversed niclosamide-stimulated ER stress (Figure 7). These results suggest that CPT induced U87 MG cytotoxicity through pathways other than ER stress.

ER stress has been reported to contribute to both autophagy and apoptosis. Niclosamide was previously observed to suppress p21 expression, which is known to induce autophagy [24]. Therefore, the present study also investigated the autophagic response after U87 MG cells were treated with niclosamide and/or CPT. Consistent with previous observations, expression of the autophagy marker LC3II was markedly increased after the U87 MG cells were exposed to niclosamide. CPT treatment also increased LC3II expression but this effect was not as potent compared with niclosamide, suggesting that CPT only serves a minor function on autophagy-induced cytotoxicity. In addition, LC3II expression displayed no clear difference between niclosamide and combined treatment (Figure 7), indicating potent autophagy activation in U87 MG cells treated with both drugs. Since the level of LC3II expression in the combined treatment associated positively with caspase-3 activation, PARP cleavage and cytochrome *c* expression, autophagy activation was likely to favor the induction of apoptosis after the U87 MG cells were treated with both CPT and niclosamide. Consistent with the results in U87 MG cells in the present study, niclosamide was reported to stimulate both apoptotic and autophagic cell death in HeLa cells [74]. In addition, a number of studies have shown that autophagy induced by anti-cancer drugs causes autophagic cell death in glioma cells [75]. Qu et al. [76] also reported that berberine-induced autophagy can reverse temozolomide resistance in glioblastoma, suggesting that autophagy upregulation can increase anticancer drug sensitivity in previously drug-resistant cancer cells. Therefore, autophagy stimulation may enhance the efficacy of anticancer drug therapy. The present study suggests that niclosamide can induce autophagy and apoptosis through ER stress, which persisted after the U87 MG cells were treated with both niclosamide and CPT together.

D-type cyclins, which include Cyclins D1, D2 and D3, are regulators of cell cycle progression [77]. Their expression has been previously associated with the prognosis in different types of cancers whilst also being used for clinical diagnosis and treatment [78–81]. Several therapeutic agents have been shown to induce cyclin D1 degradation in cancer cells. Therefore, the present study investigated effects of niclosamide and CPT on expression of D-type cyclins. Cyclin D1 and D3 were both found to be expressed in U87 MG cells, where they were suppressed after treatment with niclosamide for 6 and 24 h in U87 MG cells (Figure 8). This suggests that niclosamide can either inhibit cyclin D1 and D3 expression or induced the degradation of both. Compared with niclosamide, CPT only partially inhibited cyclin D1 expression 6 h after exposure, which returned to control levels 24 h after exposure. In addition, cyclin D3 was upregulated in response to CPT 6 h after treatment, which remained high level even after 24 h. Since cyclin D3 expression had no associations with cyclin D1, they may function independently in U87 MG cells. Cyclin D1 has been recognized to be an important human proto-oncogene, such that cyclin D1 degradation is considered to be a promising target for anticancer drugs [82]. By contrast, cyclin D3 can function as a crucial regulator of differentiation and proliferation in tumor cells [83]. It has been previously reported that cyclin D3 serves a pivotal role in the progression from G_1_ to S phase of the cell cycle, which is implicated in tumor progression. The present study revealed that niclosamide can suppress both cyclin D1 and D3 expression, suggesting that niclosamide could suppress cell proliferation by destabilizing cyclin D1 and D3. As previously documented by Shan et al. [84], ubiquitination of cyclin D1 can induce proliferation suppression in cancer cells. This may explain why increased ubiquitinated protein levels were detected following niclosamide induction in the present study.

A previous study has reported that the prognostic role of cyclin D3 in neoplasms remains controversial [85]. Activation of cyclin D3 can either promote cancer cell survival or contribute to cancer cell death. It has been reported that aberrant cyclin D3 expression was found in various cancers, including leukemia, hepatocellular carcinoma, gliomas, bladder carcinoma, prostate cancer, osteosarcoma and breast cancer [85, 86]. This abnormal cyclin D3 expression profile may reflect cell type-specific differences under distinct experimental conditions. High expression levels of cyclin D3 can sensitize cells to apoptosis and stimulate the activation of caspase-2 by direct interaction, where directed expression of cyclin D3 and caspase-2 in human cells potentiates apoptosis [87]. Olshavsky et al. [88] reported that cyclin D3 was able to bind to and attenuate androgen receptor activity in prostate cancer cells, where cyclin D3 overexpression inhibited cell proliferation in androgen-dependent cells. In addition, treatment with hydroxydibenzoylmethane, an apoptosis inducer, stimulated cyclin D3 expression in human colorectal carcinoma cells [89]. It was demonstrated for the first time in the present study that cyclin D3 expression was increased in response to CPT treatment in U87 MG cells. At present, the role of this upregulation of cyclin D3 expression by CPT remains uncertain. Since the suppression of cyclin D3 expression enhanced cytotoxicity in U87 MG cells, this may be due to an adaptive response against CPT-indued apoptosis and senescence, which may in turn be one of the mechanisms of chemoresistance induction. Therefore, combination treatment with CPT and niclosamide with cyclin D3 inhibition may serve as a novel approach as a form of adjuvant therapy for GBM.

In conclusion, findings from the present study suggested that niclosamide and CPT can exert cytotoxic effects and prevent GBM progression through different mechanisms. In addition, they appear to exhibit synergistic anticancer effects in human glioblastoma U87 MG cells. Niclosamide triggered cytotoxicity mainly through the inhibition of pro-survival signaling pathways (PI3K/AKT, Stat3 and Wnt/β-catenin) [19], induction of ER stress and autophagy. By contrast, CPT induced cell apoptosis and senescence mainly through the activation of pro-apoptotic cell signaling (MAPK/JNK), induction of p53 and cytochrome *c* expression. The combinatorial treatment of niclosamide and CPT exerted significantly more potent cytotoxic effects compared with treatment with either agent alone in U87 MG cells. Findings from a previous study [90] reported that niclosamide potentially serves a neuroprotective function against proteasome inhibition-induced apoptosis in human SH-SY5Y and rat PC12 neural cells. This may confer another advantage for selecting niclosamide as an anticancer agent for targeting glioblastoma without damaging healthy neuronal cells. Therefore, the combination of niclosamide and CPT or its derivatives may be applied to overcome chemoresistance, minimize adverse effects and treat glioblastoma in the future.

## MATERIALS AND METHODS

### Reagents

Niclosamide, CPT and PD 98059 were purchased from Sigma-Aldrich; Merck KGaA. They were dissolved in DMSO to 10 mM and stored at −20°C. MTT reagent was purchased from R&D Systems, Inc. Super Signal West Pico Plus chemiluminescence substrate was obtained from Thermo Fisher Scientific, Inc.

### Cell culture

The U87 MG cell line, which is a human glioblastoma cell line with an unknown patient origin [91], was purchased from the American Type Culture Collection (cat. no. ATCC HTB-14). Cells were cultured at 5% CO_2_ at 37°C in DMEM (Gibco; Thermo Fisher Scientific, Inc.) supplemented with 10% FBS (Corning, Inc.), penicillin (100 U/ml) and streptomycin (100 μg/ml). Cells were sub-cultured weekly onto six-well or 100-mm tissue culture dishes and used for subsequent experiments at 85–90% confluence.

### Cell treatment and viability assays

U87 MG cells were first exposed to different doses of niclosamide and CPT for 48 h. Cell viability was then measured using the MTT assay, specifically by colorimetry, according to manufacturer’s protocols. Briefly, cells (8,000 cells in 200 μl DMEM per well) were seeded into 96-well plates the day before the experiment. Cells were then treated with 1, 1.25, 2.5, 5, 10 and 20 μM niclosamide or CPT for 48 h. At the end of the treatment period, media were removed from all wells, which was then followed by the addition of 100 μl DMEM containing 1 μl MTT reagent (1:100 dilution) into each well and incubation for 2 h at 37°C. Thereafter, this media was removed and the cells were lysed using 100 μl DMSO. Absorbance in each well was then measured using a microplate reader at a wavelength of 570 nm. In total, ≥ four replicates were performed for each treatment. The concentrations for each drug were then selected for combinatorial treatment in subsequent experiments.

### Light microscopy

U87 MG cells were cultured either in the absence of treatment (control cells) or in the presence of 5 μM niclosamide and/or 5 μM CPT for 48 h in DMEM supplemented with 10% FBS at 37°C. Morphological changes of the cells were then observed and photographed under an inverted phase-contrast light microscope.

### Western blot analysis

Protein expression was measured using western blot analysis. Protein homogenates were prepared as follows: U87 MG cells were treated with 5 μM niclosamide and/or 5 μM CPT for 6–24 h. Following treatment, cells were lysed using ice-cold RIPA lysis buffer (Santa Cruz Biotechnology, Inc.) containing protease and phosphatase inhibitor cocktails (Santa Cruz Biotechnology, Inc.). Clear lysates were obtained by centrifugation at 4°C for 20 min at 40,000 g. Protein concentrations were determined using the Pierce BCA Protein Assay kit (Pierce; Thermo Fisher Scientific, Inc.) according to the manufacturer’s protocol. Subsequently, equal amounts of protein (20–30 μg) were separated by 12% or 4–20% SDS-PAGE (Bio-Rad Laboratories, Inc.), transferred onto nitrocellulose membranes and blocked for 1 h at room temperature with TBS containing 0.1% Tween 20 (TBS-T; pH 7.4) and 5% (w/v) non-fat dried milk or 5% BSA (Sigma-Aldrich; Merck KGaA). The membranes were then incubated with specific primary antibodies overnight at 4°C. Antibodies against β-actin (SC-47778), ubiquitin (SC-8017), ERK (SC-1647), cyclin D1 (SC-8396) and p53 (SC-126) were purchased from Santa Cruz Biotechnology, Inc., whereas antibodies against cleaved-PARP (9542), cleaved caspase-3 (9664), phosphorylated (p-)-ERK (4370), p-JNK (9251), p21 (64016), CCAAT/enhancer-binding protein (CHOP, 2895), LC3 (2775), cyclin D3 (2936) and cytochrome *c* (4272) were obtained from Cell Signaling Technology, Inc. After probing with primary antibodies, the membranes were washed and incubated for 1 h with corresponding HRP-conjugated secondary antibodies (SC-2357 and SC-516102, Santa Cruz Biotechnology, Inc.). The protein bands were detected using a chemiluminescent (ECL) method (Super Signal West Pico Plus chemiluminescence substrate) according to the manufacturer’s protocol. Band intensities were quantified using the ImageJ software (National Institutes of Health, version: 2.0.0-rc-43/1.52n).

### Statistical analysis

SPSS software Amos was used for statistical analysis and all data were presented as the mean ± SEM (≥ four replicates). One-way ANOVA analysis was used, followed by Tukey’s multiple comparisons test. *P* < 0.05 was considered to indicate a statistically significant difference.

## Abbreviations

GBM: glioblastoma multiforme
CPT: camptothecin
PARP: poly-(ADP ribose) polymerase
